# Assessment of cardiac oedema in patients with acute myocardial infarction by manual planimetry and computerised segmentation of triple inversion recovery prepared turbo spin echo images

**DOI:** 10.1186/1532-429X-11-S1-P51

**Published:** 2009-01-28

**Authors:** Robert I Johnstone, John P Ridgway, John D Biglands, John P Greenwood, Aleksandra Radjenovic

**Affiliations:** 1 Leeds Teaching Hospitals Trust, Leeds, UK; 2 University of Leeds, Leeds, UK

**Keywords:** Entire Left Ventricle, Steady State Free Precession Cine, Oedema Mass, Signal Intensity Threshold, Magnetisation Relaxation Time

## Introduction

The presence of oedema in human tissue increases the observed longitudinal and transverse magnetisation relaxation times (T1 and T2). MRI can therefore be used to assess the extent of cardiac oedema after a myocardial infarction (MI). The oedematous myocardium is considered to be at risk of becoming infarcted. The oedema mass in the left ventricle (LV) may therefore be a useful indicator of prognosis and of the effectiveness of treatment. Current methods of quantifying cardiac oedema are essentially visual in nature, involving either contouring and planimetry or the rating of oedema severity in cardiac segments. Inter- and intra-observer variability reduces the sensitivity of these techniques to small changes in mass.

## Purpose

To establish an automated method for the quantification of cardiac oedema and to compare the results with a visual method.

## Methods

Twenty patients (12 male, aged 41–78 [mean 61]) were scanned within 48 hours of the onset of MI symptoms and seven were rescanned four weeks later. A triple inversion recovery prepared turbo spin echo sequence was used on a Philips Intera CV 1.5 T system. 2D short-axis images of thickness 10 mm were acquired to cover the entire LV with no inter-slice gaps. Epicardial and endocardial contours were added manually to each slice image.

Oedematous regions apparent in the images were manually contoured by a cardiologist who also had access to steady state free precession cine images and late enhancement images. An automated computer segmentation was carried out using the LV contours and triple inversion recovery prepared images. The voxel intensity histogram for the entire LV was clustered by fitting two Gaussian functions to it. An initial segmentation was performed on the basis of a signal intensity threshold set at two standard deviations above the mean of the lower intensity Gaussian. Three-dimensionally connected regions with a mass less than 1 g were discarded, as were those regions that lay further than 2 mm from the endocardium. Finally, the surfaces of the regions were smoothed and holes within them were filled.

The oedema masses for the visual and computer segmentations were calculated assuming a density of 1.05 g cm^-3^.

## Results

**Figure 1:**
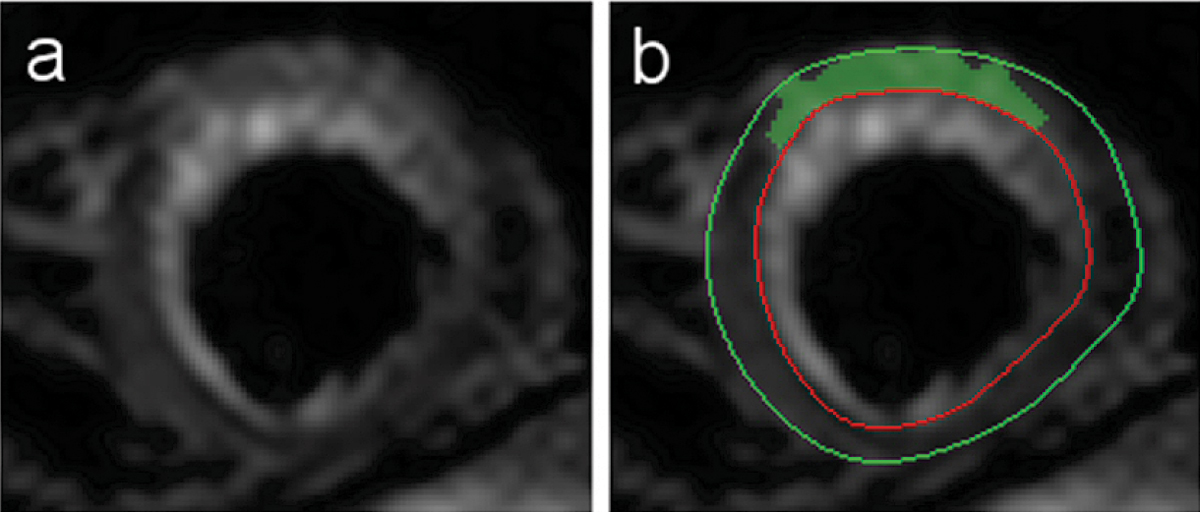
**(a) Raw slice image, (b) Slice image with LV contours added and computer segmentation shaded**.

**Figure 2:**
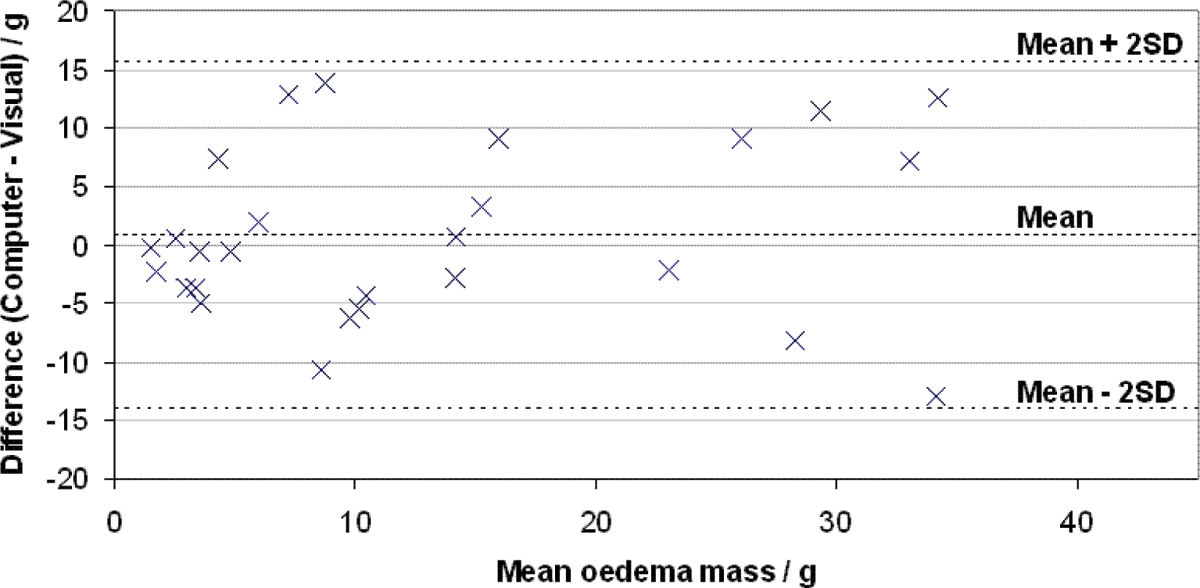
**Bland-Altman plot of differences between computer and visual segmentation**.

An example of a triple inversion recovery prepared turbo spin echo image is shown in Figure 1a. The same image slice is shown in Figure 1b with the LV contours and computer segmentation overlaid. A Bland-Altman analysis did not show statistically significant bias between the computer segmentation and the visual segmentation (+0.8 ± 2.9 g, with a 95% confidence interval). The standard deviation of the differences (SDD) was 7.4 g. The differences from the mean of the two segmentation methods are plotted against the mean in Figure 2. The mean oedema mass measured visually was 13.6 g compared to 12.8 g for the computer segmentation.

## Conclusion

The computer algorithm described has demonstrated the segmentation of cardiac oedema within images having a T2 weighting. A comparison with a visual segmentation shows no significant bias. The performance of the visual segmentation is limited by the performance of the human visual system, while the computer segmentation is hindered by its lack of anatomical and pathological knowledge. Further work is required to assess whether the computer algorithm can improve scan-rescan variability and make clinically accurate assessments of the severity and progression of cardiac oedema.

